# Bacteriophage GC1, a Novel Tectivirus Infecting *Gluconobacter Cerinus*, an Acetic Acid Bacterium Associated with Wine-Making

**DOI:** 10.3390/v10010039

**Published:** 2018-01-16

**Authors:** Cécile Philippe, Mart Krupovic, Fety Jaomanjaka, Olivier Claisse, Melina Petrel, Claire le Marrec

**Affiliations:** 1 Institut des Sciences de la Vigne et du Vin (ISVV), University Bordeaux, Equipe d’Accueil 4577, Unité de Recherche Oenologie, 33882 Villenave d’Ornon, France; cecilemphilippe@gmail.com (C.P.); jaomazava@yahoo.fr (F.J.); olivier.claisse@u-bordeaux.fr (O.C.); 2 Department of Microbiology, Institut Pasteur, 75015 Paris, France; krupovic@pasteur.fr; 3 Institut National de la Recherche Agronomique (INRA), ISVV, Unité Sous Contrat 1366 Oenologie, 33882 Villenave d’Ornon, France; 4 Bordeaux Imaging Center, University Bordeaux, Unité Mixte de Service 3420 CNRS-Unité de Service 4, Institut National de la Santé et de la Recherche Médicale, 33076 Bordeaux, France; melina.petrel@u-bordeaux.fr

**Keywords:** bacteriophage, tectivirus, wine making, must, acetic acid bacteria, *Gluconobacter cerinus*

## Abstract

The *Gluconobacter* phage GC1 is a novel member of the *Tectiviridae* family isolated from a juice sample collected during dry white wine making. The bacteriophage infects *Gluconobacter cerinus*, an acetic acid bacterium which represents a spoilage microorganism during wine making, mainly because it is able to produce ethyl alcohol and transform it into acetic acid. Transmission electron microscopy revealed tail-less icosahedral particles with a diameter of ~78 nm. The linear double-stranded DNA genome of GC1 (16,523 base pairs) contains terminal inverted repeats and carries 36 open reading frames, only a handful of which could be functionally annotated. These encode for the key proteins involved in DNA replication (protein-primed family B DNA polymerase) as well as in virion structure and assembly (major capsid protein, genome packaging ATPase (adenosine triphosphatase) and several minor capsid proteins). GC1 is the first tectivirus infecting an alphaproteobacterial host and is thus far the only temperate tectivirus of gram-negative bacteria. Based on distinctive sequence and life-style features, we propose that GC1 represents a new genus within the *Tectiviridae*, which we tentatively named “*Gammatectivirus*”. Furthermore, GC1 helps to bridge the gap in the sequence space between alphatectiviruses and betatectiviruses.

## 1. Introduction

Acetic acid bacteria (AAB) are ubiquitous, strictly aerobic bacteria occurring in sugary, alcoholic and acidic niches. They are assigned to the order *Rhodospirillales* as part of the *Alphaproteobacteria*, within the family *Acetobacteraceae*. Their metabolic potential is expressed by the partial oxidation of carbohydrates, releasing the corresponding products (aldehydes, ketones and organic acids) into the surrounding media. Several species of AAB are therefore biocatalysts for the development of eco-friendly fermentation processes as an alternative to the chemical synthesis and their oxidation machinery is exploited to produce a number of compounds such as l-ascorbic acid, dihydroxyacetone, gluconic acid and cellulose [1]. More recently the role of AAB in the production of exopolysaccharides of high molecular weight has been emphasized offering a promising alternative in the medical and food industries [2,3]. Members of the group have been also reported in a range of food and beverage ecosystems and play a positive role during the fermentation of vinegar [4], cocoa bean [5], kefir, kombucha and acidic beers [6]. In contrast, AAB represent spoilage microorganisms during wine making, mainly because they are able to produce ethyl alcohol and transform it into acetic acid. The genera *Acetobacter* and *Gluconobacter* are the most important producers of wine spoilage which is legally defined by volatile acidity, largely composed of acetic acid [7]. AAB can also produce other depreciating compounds such as ethyl acetate, acetoin from lactic acid, dihydroxyacetone and increase ropiness. Detection of AAB is reported at all stages, from the mature grape through vinification to conservation [7,8,9]. AAB originate from the leaves of the grapevine and the grapes themselves and fruit flies are considered as a common vector in propagating AAB. Different authors have observed that the less healthy the grapes, the higher the amount of AAB is [10,11]. In particular, sweet wines produced from botrytized (noble rot) exhibit significantly greater abundance of AAB compared to unaffected wines and development is in positive correlation with the *Botryotinia* sp. on grapevine leaves and in botrytized wine fermentations [12,13]. Controlled dispersion can also be guided by practices and lack of hygiene may also result in AAB being carried into the finished product. The control of AAB is challenging as these bacteria show great capacities to persist during wine making. Hence, strains of *Gluconobacter* spp. can persist at high abundance throughout wine fermentation in non-botrytized Grenache fermenting musts and a decrease in the population was only achieved at the end of alcoholic fermentation [14].

Phages infecting AAB have received relatively little attention. A few *Gluconobacter* and *Acetobacter* phages have been isolated from decaying apples [15] and also from plants where they had been causing disturbances during sorbose fermentation [16] or submerged spirit vinegar fermentations [17]. They belong to the order *Caudovirales* and possess a double-stranded DNA (dsDNA) genome enclosed in a polyhedral head, to which a tail is attached [15,16,17]. This study reports a survey of phages of AAB collected from grapes which resulted in the isolation and sequencing of a novel phage infecting *G. cerinus*, representing a novel member of the family *Tectiviridae*. Tectiviruses have tailless virions consisting of an icosahedrally organized proteinaceous capsid that surrounds a protein-rich lipid membrane, enclosing a linear double-stranded (ds) DNA genome with inverted terminal repeats and covalently attached terminal proteins [18,19,20,21,22]. Currently known tectiviruses are classified into two genera, *Alphatectivirus* and *Betatectivirus* [23]. The former genus includes 6 closely related viruses (93–98% nucleotide identity), exemplified by the bacteriophage PRD1 and infecting gram-negative bacteria of the class *Gammaproteobacteria* [24]. By contrast, members of the genus *Betatectivirus* include 6 closely related phages (67–99% nucleotide identity), represented by bacteriophage Bam35 and infecting gram-positive firmicutes of the genus *Bacillus* [25,26]. Despite close genetic relationship within each genus, members of one genus generally display no detectable sequence similarity to viruses from the other genus [27,28], suggesting an ancient divergence of the two tectivirus lineages. Furthermore, whereas all known alphatectiviruses are strictly lytic [29], betatectiviruses are temperate, persisting within their hosts as linear plasmids [30,31,32]. Nevertheless, structural studies have shown that virion architecture is conserved across the family [19,33] and is shared with many other dsDNA viruses infecting hosts in all three domains of life [34]. Based on comparative genomics and structural analyses it has been recently proposed that bacterial tectiviruses have played a key role in the evolution of many groups of big and giant dsDNA viruses of eukaryotes [35,36]. Here we describe the first tectivirus, GC1, infecting alphaproteobacterial host. GC1 is a temperate virus and displays high nucleotide sequence divergence from other known members of the family, providing an evolutionary link between the two previously known tectivirus genera, *Alphatectivirus* and *Betatectivirus*. We propose that, due to its distinctive features, GC1 should be considered as a founding member of a new genus within the *Tectiviridae*, which we tentatively named “*Gammatectivirus*”.

## 2. Material and Methods

### 2.1. Strains and Media

Strains of acetic acid bacteria (AAB) were obtained from three culture collections: ATCC, CIP and CRBO (Centre de Resources Biologiques Œnologie, Institut des Sciences de la Vigne et du Vin (ISVV), Villenave-d’Ornon, France) and included different strains of *Acetobacter aceti*, *Acetobacter pasteurianus*, *Gluconobacter cerinus* and *Gluconobacter oxydans* (Table 1). Yeast-Peptone-Mannitol (YPM) broth was used for growth of AAB. This medium contains 5 g L^−1^ yeast extract, 3 g L^−1^ peptone, 25 g L^−1^ mannitol and its pH is adjusted to pH 5. Liquid cultures were aerated for 24 h at 30 °C in a rotary shaker at 250 rpm.

### 2.2. Isolation of Acetic Acid Bacteria from Grapes

Grapes containing noble or grey rot were collected in a sterile seal plastic bag from a wine estate located in the Sauternes area near Bordeaux (France) in November 2014. These samples were transported to the ISVV and processed immediately. Grapes were hand crushed aseptically and the two obtained grape juices (named noble and grey) were immediately serially diluted in 0.85% saline. Both samples were plated on red grape juice (RGJ) agar. The medium contains per litre: 250 mL of red grape juice, 5 g of yeast extract, 1 mL of tween 80, 20 g of agar and its pH is adjusted to 4.8. It was supplemented with 0.1 mL of a 0.25% solution of penicillin (to inhibit the growth of LAB) and 0.2 mL of a 0.25% alcoholic solution of pimaricin (to suppress the growth of yeasts and moulds). After 3–5 days of incubation at 30 °C, 18 colonies from each sample were randomly picked and streaked on Glucose Yeast Extract Calcium Carbonate (GYC) agar. This medium contains 10 g L^−1^ yeast extract, 50 g L^−1^ glucose, 50 g L^−1^ CaCO_3_ and 20 g L^−1^ agar. Colonies that produced a clear halo were subjected to a catalase test. The catalase positive colonies were considered as putative AAB isolated and analysed by molecular methods.

### 2.3. AAB Identification

DNA was extracted from the bacterial isolates and stored using the FTA^®^CloneSaver™ card (Whatman^®^ BioScience, Rockville, MA, USA). DNA was used as template to be amplified by PCR with 16SDNA primers 8F and 1063R [37]. A Biorad i-Cycler was used for the amplification reactions, which were achieved in a 25 μL volume using the Taq 5× Master Mix kit (New England, Biolabs, Evry, France) and 0.2 μM of each primer. PCR conditions were 95 °C for 5 min, 30 cycles at 94 °C and 58 °C for 1 min each, 72 °C for 1.5 min and 72 °C for 7 min. Both strands of the amplified DNA of clones were sent to GATC Biotech (Konstanz, Germany) for Sanger sequencing. The gene sequences were aligned and compared with other 16S rDNA genes in the GeneBank, using the NCBI Basic Local Alignment Search Tools BLASTn program (http://www.ncbi.nlm.nih.gov/BLAST). Identification was considered valid when the identity of a contiguous sequence of 343–989 bp was at least 98%.

### 2.4. Isolation and Purification of Phages Infecting AAB

A total of 30 wine and must samples (Table 2) were centrifuged (5000× *g*, 10 min) and filtered using 0.2 μm membrane filters made of polyether sulfone. All samples were stored at 4 °C. Presence of phages was assessed using the classical double-layer plating technique [38], on YPM_Φ_ agar (YPM supplemented with MgSO_4_ 3.75 g L^−1^ and CaCl_2_ 2.375 g L^−1^). A mixture of a mid-log culture of each of the 17 indicator strains (0.2 mL at OD_600nm_ 0.2–0.3) (Table 1) and 100 µL of samples (or dilutions thereof) were added in 5 mL of molten soft-agar and poured on a bottom YPM_Φ_ plate. Plates were incubated at 30 °C for 24 h before examination of plaques. Isolated plaques were picked, suspended in 0.5 mL of sterile YPM_Φ_ medium and stored at 4 °C. They were propagated on the same strain and plaques picked again. This step was repeated twice to ensure purity.

### 2.5. Production of High-Titres Lysates

Phage lysates were prepared by culturing *G. cerinus* CRBO11179 to an OD_600_ of 0.2 and addition of phage particles at a multiplicity of infection (MOI) of 0.003. Infected cultures were incubated at 30 min for adsorption and shaken until lysis was observed. The sample was centrifuged and the supernatant was filtered. After repeated rounds of phage multiplication, phage titres ranging from 10^8^ to 10^10^ Plaque Forming Unit (PFU) mL^−1^ were obtained and stored at 4 °C until use.

### 2.6. Chloroform Sensitivity Assay

GC1 phage samples (2 mL) were incubated with and without various volumes of chloroform (up to 80 μL) in capped glass tubes with gentle mixing at room temperature for 15, 30 and 60 min. The titres of the mixtures were then determined on *G. cerinus* CRBO11179.

### 2.7. Concentration of Phage

To obtain highly concentrated phage preparations, 500 mL of phage lysate was treated with DNase I (1 μg mL^−1^; Invitrogen, Illkirch, France) and RNase A (1 μg mL^−1^; Promega, Charbonnières, France) at 37 °C for 30 min. Polyethylene glycol (PEG) precipitation was carried out as follows. Sodium chloride (final concentration, 1 M) and PEG 6000 (10%, *w*/*v*) were added to the treated lysate. The sample was stored overnight at 4 °C and sedimented 10 min at 11,000× *g* at 4 °C. The pellet was carefully resuspended in a small volume of Tris Magnesium (TM) buffer (50 mM Tris-HCl, MgS0_4_ 10 mM), resulting in a 25-fold concentration.

### 2.8. Electron Microscopy

Phages (10 µL; ~10^10^ mL^−1^) were deposited on carbon-coated copper grids (200 mesh) for 30 s and coloured with uranyl acetate (saturated in water, pH 4.5 for 30 s). Stained particles were examined with a Hitachi H7650 electron microscope operated at 80 kV.

### 2.9. Dynamic Light Scattering (DLS)

The particle size of GC1 was measured by dynamic light scattering (DLS) using a Zetasizer Z/S system (Malvern, UK) at 37 °C. Cuvettes filled with the sample were carefully inspected to avoid air bubbles. Phages were diluted in distilled water (dH_2_O) to a final concentration of 10^8^ PFU mL^−1^. Measurements were repeated at least three times.

### 2.10. Extraction of Phage DNA and Restriction Digestion

A purified lysate (25 mL, titre ≥ 10^8^ PFU mL^−1^) was added with PEG (10%) as previously described. After one night at 4 °C, the sample was centrifuged (9000 rpm for 30 min at 4 °C). The pellet was suspended in 500 µL of TM buffer and treated with DNAse and RNAse (1 µg mL^−1^) at 37 °C for 30 min. Phage DNA was first purified using the standard method used in our laboratory to extract oenophage DNA (phenol chloroform method) [38,39,40]. However, the method failed in extracting the GC1 DNA. The nucleic acid could only be extracted following denaturation of the viral coat proteins using proteinase K and treatment with a higher concentration of SDS as described previously to purify DNA from *Streptococus thermophilus* bacteriophages [41].

Purified phage DNA was resuspended in 50 µL of Tris–EDTA buffer (pH 7.6) and quantified by optical density at 260/280 nm with a Biospec-nano spectrophotometer (Shimadzu, Columbia, MD, USA). DNA was digested with the restriction endonucleases *Eco*RI, *Eco*RV and *Nde*I under the conditions recommended by the manufacturer (New England Biolabs, Evry, France). Restricted DNA was electrophoresed on 0.8% (*w*/*v*) agarose gels in 1× TAE buffer (40 mM Tris-acetate, 1 mM EDTA) and visualized by UV photography after staining with ethidium bromide. The 100-bp DNA ladder (PM) (New England Biolabs, Evry, France) was used as marker. The size of the phage genome was estimated by summing up of the lengths of restriction fragments.

### 2.11. DNA Sequencing

Phage GC1 was sequenced at the CGFB platform (Centre Génomique Fonctionnelle de Bordeaux) using the Truseq kit (Illumina, San Diego, CA, USA) and an Illumina Miseq system with 2 × 250 bp paired-end reads. Assembly of the resulting reads was performed using SPAdes version 3.10.1 [42]. The inverted terminal repeats were amplified by PCR (Table 3) and sequence determination obtained by Sanger sequencing (Eurofins MWG Operon, Les Ullis, France). Structural and functional annotations were obtained from Prokka [43]. Genome alignments were performed using EasyFig [44]. Complete sequences of the *Salmonella* bacteriophage PRD1 and *Bacillus* phage Bam35c (GenBank accession numbers: NC_001421 and PRJNA14311, respectively) were used for comparison. The nucleotide sequence determined in this study was submitted to the GenBank database under accession numbers MG159787.

### 2.12. Bacterial Lysogen Generation

GC1 was propagated on strain CRBO11179. Two single turbid plaques were randomly selected, picked with sterile toothpicks and isolated on YPM agar plates. Five colonies were randomly selected from each plate and examined for lysogeny by looking for immunity to infection. A GC1 lysate was spotted on overlays of the 10 putative lysogens. The absence of plaques was taken as an indication of immunity. Two resistant clones (one from each original plaque) were subsequently grown in YPM broth and the presence of spontaneously released viruses in supernatant was detected by the double-layer plate method. Chromosomal DNA was extracted and screened by PCR with primers ITR-R and gp1-F to confirm positive tectiviral signals.

### 2.13. Homology Searches and Phylogenetic Analyses

The *in silico*-translated protein sequences were used as queries to search for sequence homologs in the non-redundant protein database at the National Centre for Biotechnology Information using BLASTP [45] with an upper threshold E value of 1 × 10^−3^. Searches for distant homologs were performed using HHpred [46] against different protein databases, including PFAM (Database of Protein Families), PDB (Protein Data Bank), CDD (Conserved Domains Database) and COG (Clusters of Orthologous Groups), which are accessible via the HHpred website. Searches against the CDD database at NCBI were also performed using CD-search [47].

For phylogenetic analyses, sequences were aligned using PROMALS3D [48] and uninformative positions removed using the gappyout function of trimAL program [49]. Maximum likelihood phylogenetic trees were constructed using the PhyML [50], the latest version of which includes automatic selection of the best-fit substitution model for a given alignment. The best models were identified by PhyML as follows: LG + G (ATPase); RtREV + G + F (MCP); VT + G + I + F (DNAP). A Bayesian-like transformation of aLRT (aBayes), as implemented in PhyML [50] was used to estimate branch support. Pairwise intergenomic distances between all sequenced tectiviruses were calculated with the Genome-BLAST Distance Phylogeny (GBDP) method under settings (distance formula *d*_6_) recommended for prokaryotic viruses using VICTOR tool [51].

## 3. Results and Discussion

### 3.1. Isolation of GC1 from Enological Samples

**Figure 1:**
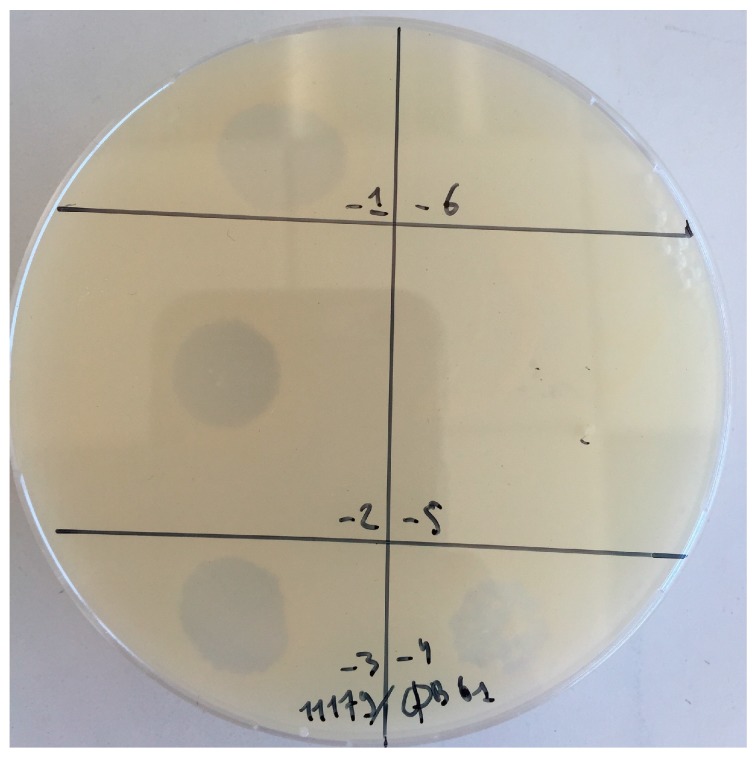
Plaques produced by phage GC1 on *G. cerinus* ATCC 11179.

We explored the presence of phages infecting AAB in 30 enological samples. As shown in Table 2, the samples corresponded to musts, PDC and wines collected during the 2013 and 2014 vintages from various wineries, making red, dry white and sweet Bordeaux (Sauternes) wines [38]. Each of the 30 samples was tested against a panel of 17 isolates belonging to the two main genera of AAB associated with wine making, namely *Gluconobacter* and *Acetobacter* (Table 1). During the survey, a juice sample collected during dry white wine making, produced a few plaques on strain *Gluconobacter cerinus* CRBO11179. A single plaque was subsequently isolated and a pure stock of phage GC1 was prepared following three successive rounds of plaque purification. A high titre phage stock was prepared by liquid infection in YPM_Φ_ broth. Optimal infection was observed at an MOI value of 0.003 and the phage titres as high as 10^10^ PFU mL^−1^ in the lysate were obtained after 6 h of incubation at 30 °C. As seen in Figure 1, phage GC1 produced turbid plaques on its host.

### 3.2. Host Spectrum

The GC1 high titre lysate was used to perform a host range test. We first confirmed that GC1 could not infect any of the 16 strains used during the initial survey, including 3 isolates of *G. cerinus* and 4 isolates of each of the following species: *Gluconobacter oxydans*, *Acetobacter aceti* and *Acetobacter pasteurianus*.

Mateo and colleagues [11] recently observed that *G. cerinus* was dominantly recovered from grapes, especially in mould-infected and rot-affected grapes. To find additional hosts for GC1, grapes containing grey (G) and noble (N) rot were collected in Sauternes (France) and screened for AAB. A total of 36 colonies grown on GY were randomly selected and their sensitivity towards GC1 assessed. The phage was able to infect two isolates recovered from noble rot (N8 and N11) and the infection was as productive as the one observed with strain CRBO11179 (efficiency of plating [eop] of 1). Sequencing of 16S rDNA showed that both isolates also belonged to the *G. cerinus* species (100% identity). Thus, our results show that the host spectrum of GC1 is likely to be limited to strains belonging to the *G. cerinus* species. However, it is noteworthy that although the phage has been isolated from a juice sample collected during dry white wine making, it is active against strains associated with other wine types: a red wine for strain CRBO11179 and sweet wine for N8 and N11.

### 3.3. Morphology and Genome Analysis

**Figure 2:**
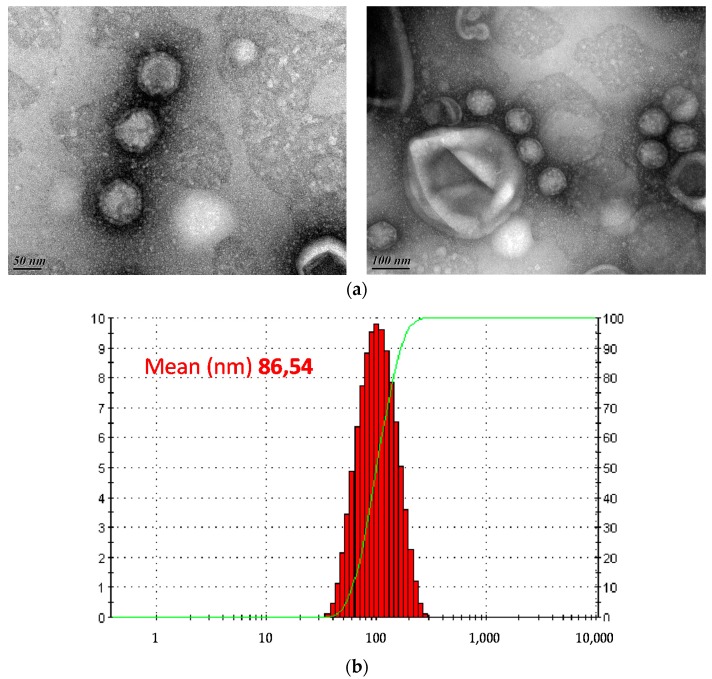
Morphology and size of BG1 particles. (**a**) Transmission electron micrographs of phage GC1. Scale bars are 50 nm (left) and 100 nm (right); (**b**) Average size (nm) of the particles in dynamic light scattering (DLS) analysis, using a Zetasizer Z/S system (Malvern, UK).

Transmission electron microscopy revealed tail-less icosahedral particles, which displayed a diameter of c. 78 nm in diameter (Figure 2a). Accordingly, the diameter measured using dynamic light scattering was 85 nm (Figure 2b). The absence of a visible tail structure suggested that GC1 is not a member of the order *Caudovirales*, the most common type of bacterial viruses [52] but instead could belong to either one of the three families of tail-less bacteriophages with isometric capsids, namely *Tectiviridae* [18], *Corticoviridae* [53] or *Sphaerolipoviridae* [54].

**Figure 3:**
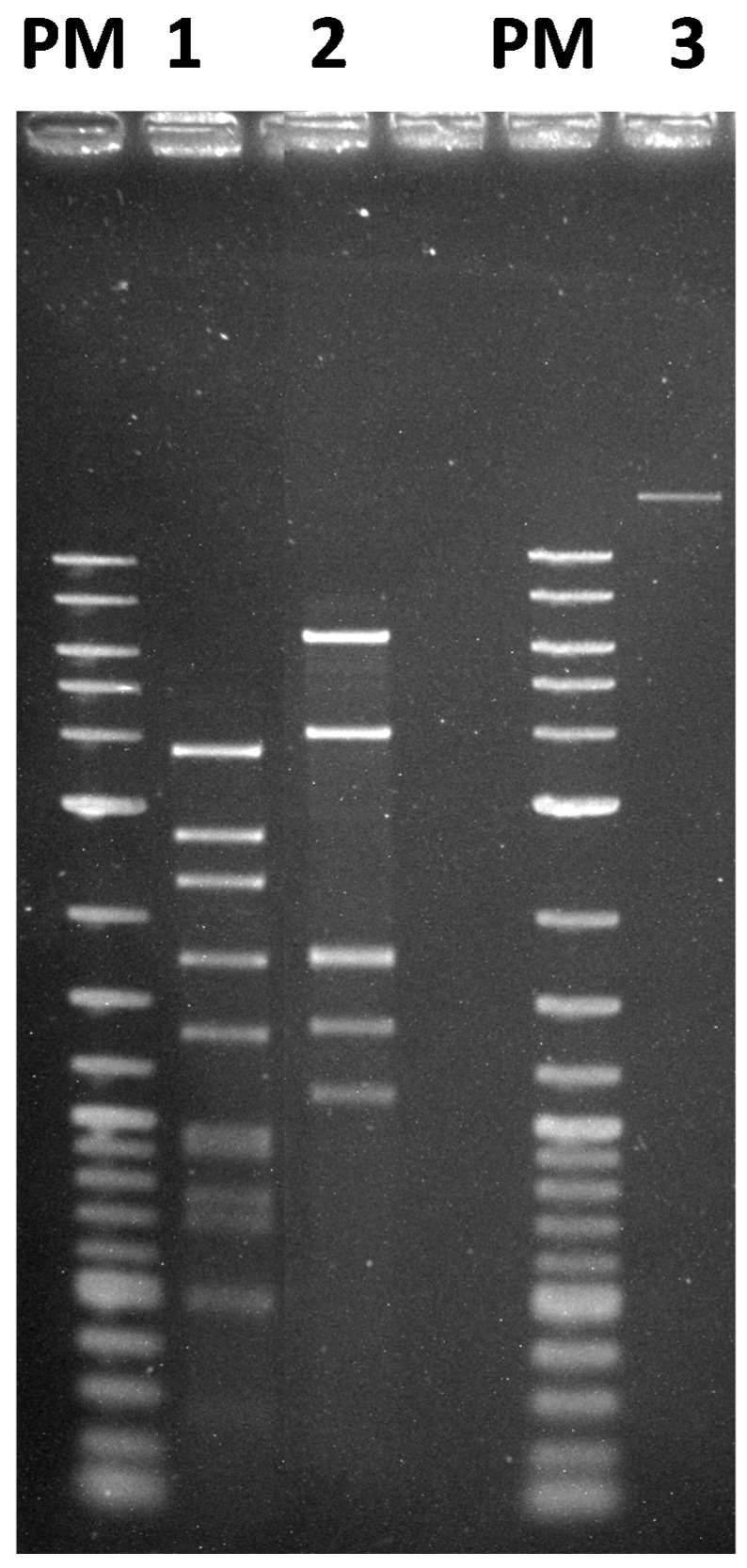
Restriction enzyme patterns of phage GC1 DNA. Lane 1, *Eco*RI; lane 2, *Eco*RV, lane 3, undigested DNA. PM, 2-Log DNA ladder (Biolabs, Ipswich, MA, USA).

To gain a more ample understanding of the phage, nucleic acid was extracted from the GC1 viral particles. The extracted nucleic acid could be digested with different type II restriction endonucleases, including *Eco*RI, *Nde*I and *Eco*RV, showing that GC1 has a double-stranded *DNA* (dsDNA) genome (Figure 3). The average size of the genome estimated from the sum of DNA fragments generated with the three restriction endonucleases was 17 kb and the genome did not contain a *cos* site.

Based on the data obtained through sequencing, the genome of GC1 could be assembled into one final contig that comprises 16,523 bp, which is in reasonable agreement with our first estimated value. The virtual digest of the assembled contig with *Eco*RI and *Eco*RV matched the restriction profiles of the phage DNA digested with the same enzymes, confirming the proper assembly. These results indicate that phage GC1 has a small dsDNA linear genome with inverted terminal repeats (ITR) of 326 bp at both termini. Two pairs of primers specific to each extremity of the linear genome were designed (Table 3) and used to confirm that the ITRs are indeed adjacent to ORF1 and ORF36, respectively. Collectively, the morphological and genomic characteristics of GC1 suggest that it belongs to the family *Tectiviridae*. Notably, the genome of GC1 is the largest among known tectiviruses, none of which exceeds 15 kb.

### 3.4. Virion Stability

**Figure 4:**
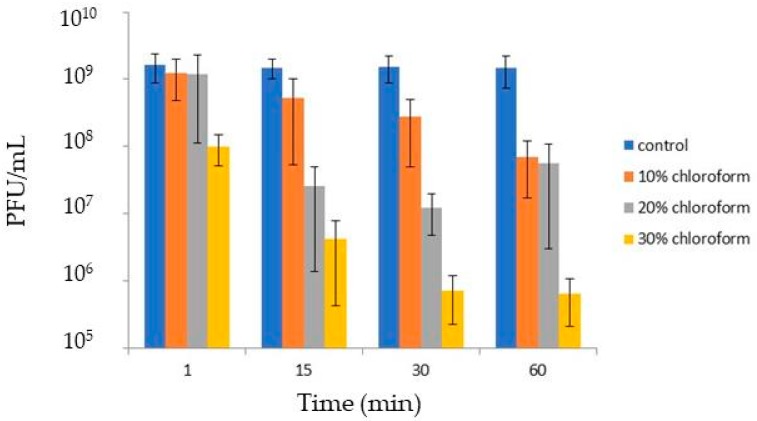
Sensitivity of GC1 to chloroform. The phage lysate was added with chloroform (10%, 20% or 30%). After incubation for 1, 15, 30 or 60 min at room temperature, PFU were enumerated.

Tectiviruses have a lipid bilayer beneath the icosahedral protein shell which is formed of approximately equal amounts of virus-encoded proteins and lipids derived from the host cell plasma membrane [55]. Upon infection, the viral membrane transforms into a tail-like structure, which plays a crucial role in the delivery of the viral genome into the host cell by forming a tubular structure that penetrates the cell envelope [56,57]. To test if GC1 virions contain lipids, the original lysate was treated with different concentrations of chloroform. Figure 4 shows that the solvent reduced virus infectivity in a concentration and time dependent manner. The phage was not completely inactivated and increasing the CHCl_3_ concentration to 30% produced the stronger reduction (3 log) within 30 min. Our results are therefore consistent with GC1 possessing an inner lipid membrane. The sensitivity of other reported tectiviruses such as Wip1, Bam35 and GIL16 to chloroform has been tested in the past. However different protocols have been used to prepare the lysates making comparisons difficult. We also observed that incubation of the GC1 lysate at 4 °C for three months decreased the virus titre by 1 log. Taken together the results suggest that GC1 virions are physically robust.

### 3.5. ORF Function Assignment and Genomic Organization

**Figure 5:**
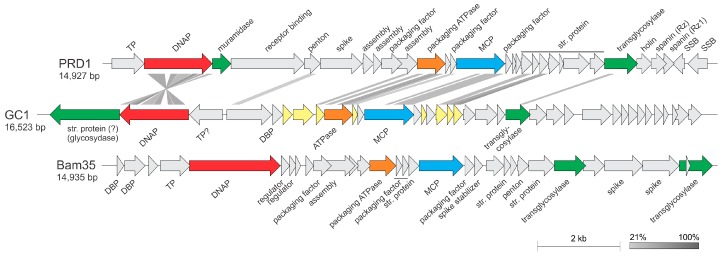
Genome maps of tectiviruses GC1, PRD1 and Bam35. The figure was generated using EasyFig [44] and shows the similarity between translated nucleotide sequences as determined by the tblastx algorithm. Abbreviations: TP, terminal protein; DNAP, family B DNA polymerase; MCP, major capsid protein; str. Protein, structural protein; SSB, single-stranded DNA-binding protein; DBP, DNA-binding protein. ORFs shared with alphatectiviruses are highlighted in light yellow (see Table S1 for annotations). ORFs filled in grey do not have homologs in either of the other two genomes. The color code for gene function is DNAP (red), muramidase (green), packaging ATPase (orange), MCP (blue), transglycosylase (green).

Analysis of the GC1 genome sequence revealed a total of 36 open reading frames (ORFs), most of which (24) were smaller than 100 codons. The vast majority of ORFs, 33 (92%), are found on the forward strand and 3 (8%) are found on the opposite strand (Figure 5). The molecular G + C content of the GC1 genome is slightly lower than that of the first sequenced *G. cerinus* isolate (50.5% versus 55.68%, respectively) [58].

Homology searches were performed using a combination of BLASTP [45] as well as a more sensitive hidden Markov model (HMM)-based HHpred program [46] and the CD-search against the Conserved Domains Database at NCBI [47]. Searches using the BLASTP program revealed that only eight (22%) of GC1 gene products are significantly similar (cut-off of E = 1 × 10^−3^) to sequences in the non-redundant protein database (Table S1). The closest homologs for five of these proteins (ORFs 2, 9, 12, 16 and 17) were encoded by members of the genus *Alphatectivirus* (*Tectiviridae*). These included the key proteins involved in DNA replication (protein-primed family B DNA polymerase (DNAP)) as well as in virion structure and assembly (major capsid protein, genome packaging ATPase and two minor structural proteins). Considering the short length of most of the GC1 proteins, the BLASTP search database was restricted to the family *Tectiviridae* (taxid: 10656). This adjustment resulted in identification of five additional putative homologs conserved between GC1 and alphatectiviruses (Table S1). The homologous genes occupied equivalent positions within the GC1 genome when compared to the genomic layout of other tectiviruses, with a notable inversion of the region encompassing the genes for the DNAP and putative terminal protein (Figure 5), which in viral genomes is typically encoded immediately upstream of the DNAP [59].

Several GC1 proteins with no orthologues in known tectiviruses have been apparently recruited from diverse sources in the course of adaptation to the ecological niche or the host. For instance, ORF1 encodes a putative glycosydase, found in the tail-spike proteins of various tailed bacteriophages, where the glycosydase domain is responsible for depolymerization of the capsular polysaccharide during virus entry [60]. Indeed, HHpred analysis identified the tail-spike proteins of bacteriophages HK620, LKA1, phi297 and K1-5 as homologs of the GC1 ORF1 with high probability (*p* > 99%). ORF1 in GC1 genome is located transcriptionally downstream of the DNAP gene (Figure 5). Notably, the corresponding position in other alphatectiviruses is occupied by a non-homologous but functionally equivalent gene encoding a muramidase (Figure 5), which in bacteriophage PRD1 is a structural component of the virion [61]. It is thus likely that the product of ORF1 represents a structural component of the GC1 virion assisting in virus penetration through the host cell envelope. Closest homologs of ORF1 are encoded by various *Alphaproteobacteria*, including various *Gluconobacter* species, suggesting that ORF1 has been acquired by GC1 by horizontal gene transfer from its host.

Besides the above mentioned muramidase, most tectiviruses encode a second muralytic enzyme, a transglycosylase, which is also a structural component of the virion in PRD1-like alphatectiviruses [61]. HHpred analysis has shown that ORF22 is a divergent transglycosylase (*p* = 99%; Table S1), which is encoded in the equivalent genomic locus as those of other tectiviruses (Figure 5). Interestingly, however, the protein does not display detectable sequence similarity to the corresponding proteins of tectiviruses but has closest homologs in bacteria, suggesting that during the evolution of tectiviruses the transglycosylase gene has undergone a non-orthologous replacement. Interestingly, ORF23 encodes a homolog of a structural protein of the corticovirus PM2, which has been previously noted to display a promiscuous distribution in various bacteriophages, including members of the order *Caudovirales* [62].

### 3.6. GC1 Is a Temperate Phage

Whereas alphatectiviruses are strictly lytic, betatectiviruses, such as the *Bacillus thuringiensis* phage GIL01, are temperate. However, they do not integrate into the bacterial chromosome but instead, replicate independently within their host and occasionally experience induction of their lytic cycle. This prompted us to evaluate whether the turbid plaques produced by phage GC1 on *G. cerinus* could suggest lysogeny. Ten putative lysogenic clones were isolated from the centres of two turbid plaques. They were examined for lysogeny by looking for immunity to infection and six clones did not support GC1 propagation (plaque assays). Two clones named L3 and L6 were further selected and were shown to release small amounts of virus into culture supernatants. The purified DNAs from strains L3 and L6 were tested in a PCR assay with primers ITR-R and gp1-F which confirmed positive tectiviral signals. Consequently, L3 and L6 are lysogenic strains carrying GC1 prophage.

### 3.7. Phylogenetic Position of GC1 within the TECTIVIRIDAE

**Figure 6:**
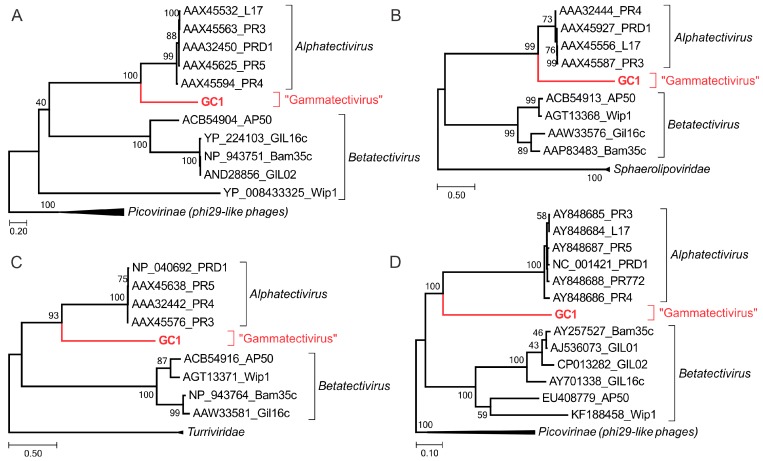
Phylogenetic relationship of GC1 compared with other tectiviruses based on the three conserved proteins: the major capsid protein (**A**); packaging ATPase (**B**) and DNAP (**C**); Phylogenetic tree computed using the Genome BLAST Distance Phylogeny (GBDP) strategy (**D**); Maximum likelihood phylogenetic trees (**A**–**C**) were computed using the best-fit substitution models for given alignments (see Materials and Methods), as determined by PhyML [50]. The trees were rooted with taxa including the closest homologs of the corresponding proteins outside of the family *Tectiviridae*. Scale bars in (**A**–**C**) represent the number of substitutions per site, whereas in panel (**D**); the branch lengths are scaled according to the distance formula (*d*_6_) used for the calculation, i.e. sum of all identities found in high-scoring segment pairs between the two proteomes divided by total genome length.

To explore the position of GC1 within the family *Tectiviridae*, we performed maximum likelihood phylogenetic analysis of the three key proteins—MCP, packaging ATPase and DNAP—conserved in all members of the family. In all cases, GC1 formed a well-supported sister group to the closely related PRD1-like tectiviruses from the genus *Alphatectivirus* (Figure 6a–c). Next, we calculated pairwise intergenomic distances between all sequenced tectiviruses with the Genome-BLAST Distance Phylogeny (GBDP) method using VICTOR tool [51]. This genome-wide comparison also placed GC1 as a deep-branching sister group to PRD1-like tectiviruses (Figure 6d). Consistently, sequence analysis of the GC1 gene products (Figure 5, Table S1) suggest that the virus is most closely related to PRD1-like phages of the genus *Alphatectivirus*. However, unlike other alphatectiviruses, GC1 is a temperate phage. Furthermore, pairwise comparison of GC1 with other alphatectiviruses using BLASTN, as recommended by the ICTV (International Committee of Viruses) Bacterial and Archaeal Viruses Subcommittee (PMID: 26733293), did not reveal common regions of sufficient similarity at the nucleotide level. Collectively, these results confirm that GC1 is a highly divergent member of the family *Tectiviridae*, which cannot be placed into either existing genus based on the recommended demarcation criteria [23]. Thus, we propose that GC1 represents a new genus within the *Tectiviridae*, which we tentatively named “*Gammatectivirus*”. Interestingly, GC1 helps to bridge the gap in the sequence space between alphatectiviruses and betatectiviruses. For instance, in a PSI-BLAST search seeded with the MCP sequence of GC1, all alphatectiviruses and betatectiviruses were recovered after three iterations (E < 0.01), whereas the leap to betatectiviruses was not possible when the search was seeded with the MCP sequence of PRD1-like phages.

## 4. Conclusions

During a survey of bacteriophages infecting wine-associated AAB, we identified and characterized bacteriophage GC1, a founding member of the new proposed genus “*Gammatectivirus*”. GC1 displays unique life-style features among tectiviruses infecting gram-negative bacteria. For instance, we could demonstrate that GC1 enters a lysogenic state in *G. cerinus*. The influence of temperate betatectiviruses GIL01 and GIL16 on certain life traits of *B*. *thuringiensis* serovar israelensis has been recently investigated [63]. Lysogeny was shown to have a significant influence on the bacterial growth, sporulation rate, biofilm formation and swarming motility of *B*. *thuringiensis*, all of which are traits involved in the survival and colonization of this bacterium in different environmental habitats. Studies are now in progress to assess the role of such interactions in the persistence of AAB during wine making. Because *G. cerinus* is a commensal bacterium of the gut of *Drosophila melanogaster* [64], future work should also consider the role of lysogeny in the interactions between gut-associated microbes of *Drosophila melanogaster* [65] and possible influence on the fruit fly’s behaviour [66].

## Figures and Tables

**Table 1:** Strains used in the study.

Strains	Name	Isolation	
		Year	Source
*Acetobacter aceti*	CRBO08ba14	2008	Grapes, Aquitaine
	CIP103111 *	na	Alcohol vinegar
	ATCC15973 *	na	Wine vinegar
*Acetobacter pasteurianus*	CRBO03ba01, CRBO03ba02	2003	na
	CRBO08ba01, CRBO08ba03	2008	Grapes, Aquitaine
*Gluconobacter cerinus*	CRBO11174, CRBO11176, CRBO11178, CRBO11179	2011	Matured grapes, Grande Ferrade, INRA Bordeaux
	CRBO96ba02	1996	Grapes, Pessac-Leognan
	CRBO96ba39	1997	Matured grapes, Bordeaux (La Tour Blanche)
*Gluconobacter oxydans*	CRBO11187	2011	Matured grapes, Bordeaux
	CRBO11203, CRBO11204, CRBO11205	2011	Matured grapes, Léoville

* Type strain; na, non available.

**Table 2:** Global survey of phages infecting AAB in 30 samples of oenological origin.

Number	Samples Characteristics ^a^			Presence of Phages ^b^
	Wine	Appellation/Grape Variety	Step	
67, 68, 69, 70	Sweet white	Sauternes/Sémillon	Must	−
14			PDC	−
12	Dry white	Bordeaux/Sémillon	PDC	−
1		Bordeaux/SauvBlanc	Must	−
2			Juice for PDC	+
26			PDC	−
27, 29			Early AF	−
31		Lussac Saint Emilion/SauvBlanc	PDC	−
125	Red	Saint-Emilion/CabFranc		−
18		Saint Emilion/Merlot	Must	−
62				−
3		Lussac Saint Emilion/Merlot	Must	−
30				−
22, 23, 24			PDC	−
60, 63		Margaux/Merlot	Must	−
61		Entre-Deux-Mers/Merlot	Must	−
143		Pomerol/CabSauv	MLF	−
144, 147		Pomerol/Merlot		−
148		Gers-Gamay/Merlot		−
149		Gers/Merlot		−
151, 153		Medoc/CabSauv		−

^a^ The 30 samples were obtained from a collection of 166 samples (numbered 1 to 166) as previously reported [38]. They were provided by different wine estates located in the Bordeaux area during the 2013 and 2014 vintages and collected at different steps during wine making (PDC, pied de cuve; AF, alcoholic fermentation; MLF, malolactic fermentation). They were previously shown to contain active phages against the lactic acid bacterium *Oenococcus oeni* [38]. ^b^ Each of the 30 samples was tested against a panel of 17 isolates belonging to the two main genera of AAB associated with wine making, namely *Gluconobacter* and *Acetobacter* and presence/absence of plaques is indicated (+/−). Plaques identified in sample 2 were observed on *G. cerinus* CRBO11179.

**Table 3:** Primers used in the study.

Name	Sequence (5′-3′)	Position on GC1-Genome
*ITR-F*	CTCTTCCACGGCAACAATCC	271–252 16,253–16,272
*ITR-R*	ACAAGTACTACAGGGAGGGG	52–71 16,472–16,453
*gp36-F*	TAAGCGCGGATGGTTTAAGC	15,957–15,976
*gp1-F*	TACAATCGTGACGGCGGATA	886–867

Primers ITR-R and gp1-F amplified a 835 bp amplicon (left ITR). Primers ITR-R and gp36 F amplified a 516 bp amplicon (right ITR) (see Figure 5).

## Supplementary Materials

The following are available online at www.mdpi.com/1999-4915/10/1/39/s1, Table S1. Summary of the general features of *G. cerinus* phage GC1.
