# Supplementary material for: Bacteriophage GC1, a Novel Tectivirus Infecting Gluconobacter Cerinus, an Acetic Acid Bacterium Associated with Wine-Making
Source: Viruses. 2018 Jan 16;10(1):39. doi: 10.3390/v10010039 (PMC5795452; doi:10.3390/v10010039)
Supplement: Supplementary file 1 [file viruses-10-00039-s001.pdf]

Table S1. Summary of the general features of *G. cerinus* phage GC1

N° CDS	Size(aa)	Strand	Putative function	Conserved domain	BBH (Source, accession)	identical/total; % id	e-value
1	559	-	Hypothetical protein	rhamnogalacturonase, glycosydase domain (1RMG, P=99.6), likely receptor-binding protein	Hypothetical protein (*Gluconobacter oxydans*, WP_062494220.1)	135/529; 26	2e-29
2	561	-	DNA polymerase	DNA_pol_B_2 (pfam03175)	DNA polymerase (phage PRD1, NP_040682.1)	235/571; 41	1e-130
3	271	-	Hypothetical protein	-	-	-	-
4	367	+	Hypothetical protein	-	-	-	-
5	86	+	Hypothetical protein	HTH DNA-binding protein (3F8M, P=90)	-	-	-
6	74	+	Hypothetical protein	-	Assembly protein PRD1-P10, fragment (phage PRD1, gi|61394104|AAX45904.1)	26/61; 43	0.03
7	169	+	Hypothetical protein	-	Packaging efficiency factor PRD1-P6 (Enterobacteria phage L17, AAX45553)	36/116; 31	0.003
8	75	+	Hypothetical protein		Assembly protein PRD1-P10, fragment (Enterobacteria phage PR4, AAA32443)	28/65; 43	4e-08
9	225	+	DNA packaging ATPase	-	DNA packaging ATPase PRD1-P9 (phage PRD1, NP_040689.1)	95/231; 41	2e-56
10	40	+	Hypothetical protein	-	PRD1-P10 (Salmonella virus PRD1, NP_040690)	11/31, 35	5e-04
11	46	+	Hypothetical protein	-	-	-	-
12	399	+	Major capsid protein	Phage_Capsid_P3 (pfam09018)	Chain A, P3, The Major Coat Protein (phage PRD1, 1HX6_A)	120/377; 32	8e-43
13	63	+	Hypothetical protein	-	-	-	-
14	38	+	Hypothetical protein	-	PRD1-gpx (Salmonella virus PRD1, YP_001542610)	10/16, 63	0.019
15	63	+	Hypothetical protein	-	-	-	-
16	87	+	Hypothetical protein		DNA delivery protein, PRD1-P18 (phage PRD1, gi|61394111|AAX45911.1)	25/85; 29	6e-10
17	55	+	Hypothetical protein	PRD1_DD phage membrane DNA delivery (pfam11087)	DNA delivery protein, PRD1-P32 (phage PRD1, YP_001542612)	28/53; 53	1e-07
18	71	+	DNA delivery protein	PRD1_DD phage membrane DNA delivery (pfam11087)	DNA delivery protein, PRD1-P34 (phage PRD1, NP_040696)	31/53; 58	3e-16
19	96	+	Hypothetical protein	-	-	-	-
20	180	+	Hypothetical protein	-	-	-	-
21	61	+	Hypothetical protein	-	-	-	-
22	199	+	Peptidase	Lytic transglycosylase (4C5F, P=99.4)	hypothetical protein (*Paenibacillus herberti*, WP_089524389.1)	33/77; 43	8e-04
23	135	+	Structural protein	Virion protein (PHA02564)	Structural protein (*Klebsiella aerogenes*, WP_032708580.1)<br>Structural protein P5 (Pseudoalteromonas phage PM2, NP_049909)	60/130; 46<br>49/133; 37	4e-30<br>3e-19
24	137	+	Hypothetical protein	-	-	-	-
25	76	+	Hypothetical protein		-	-	-
26	225	+	Hypothetical protein	-	-	-	-
27	68	+	Hypothetical protein		-	-	-
28	55	+	Hypothetical protein	-	-	-	-
29	81	+	Hypothetical protein	-	-	-	-
30	59	+	Hypothetical protein	-			

N° CDS	Size(aa)	Strand	Putative function	Conserved domain	BBH (Source, accession)	identical/total; % id	e-value
31	57	+	Hypothetical protein	-	Hypothetical protein OR214_00015 (*Ralstonia pickettii* OR214, ENZ79599.1)	18/39; 46	0.01
32	69	+	Hypothetical protein	-	-	-	-
33	45	+	Hypothetical protein	-	-	-	-
34	38	+	Hypothetical protein	-	-	-	-
35	92	+	Hypothetical protein	-	-	-	-
36	97	+	Hypothetical protein	-	Hypothetical protein (*Acetobacter persici*, WP_077932138.1)	25/106; 24	0.017
